# A novel approach to wireless electromagnetic tracking using frequency modulation radio communication

**DOI:** 10.1007/s11548-023-02981-4

**Published:** 2023-06-29

**Authors:** Daragh Crowley, Marco Cavaliere, Pádraig Cantillon-Murphy

**Affiliations:** 1 Tyndall National Institute, Lee Maltings, Dyke Parade, Cork, Ireland; 2 School of Engineering, University College Cork, College Rd, Cork, Ireland

**Keywords:** Electromagnetic tracking, Wireless sensing, Surgical navigation, RF communication

## Abstract

**Purpose:**

Electromagnetic tracking (EMT) is beneficial in image-guided interventions to reduce the use of ionising radiation-based imaging techniques. Enabling wirelessly tracked sensors will increase the usability of these systems for catheter tracking and patient registration systems. This work introduces a novel method of wirelessly transmitting sensor data using a frequency modulation (FM) radio.

**Methods:**

The proposed technique was tested using the open-source Anser EMT system. An electromagnetic sensor was connected in parallel to an FM transmitter prototype and wired directly to the Anser system for comparison. The performance of the FM transmitter was evaluated on a grid of 125 test points using an optical tracking system as a gold standard.

**Results:**

An average position accuracy of 1.61 ± 0.68 mm and angular rotation accuracy of 0.04° for the FM transmitted sensor signal was obtained over a 30 cm × 30 cm × 30 cm volume, in comparison with the 1.14 ± 0.80 mm, 0.04° accuracy previously reported for the Anser system. The FM transmitted sensor signal had an average resolved position precision of 0.95 mm while the directly wired signal was found to have an average precision of 1.09 mm. A very low frequency ($\sim$ 5 mHz) oscillation in the wirelessly transmitted signal was observed and compensated for by performing a dynamic scaling of the magnetic field model used for solving the sensor pose.

**Conclusions:**

We demonstrate that FM transmission of an electromagnetic sensor signal can be used to achieve similar tracking performance to a wired sensor. FM transmission for wireless EMT is a viable alternative to digital sampling and transmission over Bluetooth. Future work will create an integrated wireless sensor node using FM communication that is compatible with existing EMT systems.

## Purpose

Electromagnetic tracking (EMT) can potentially reduce exposure to harmful ionising radiation during image-guided interventions by providing accurate tracking of surgical tools inside the body without requiring line of sight [1]. The wired sensors used in currently available EMT systems may prove inconvenient in clinical applications requiring many sensors. A wireless EMT sensor suitable for integration in an endoscope or patient-mounted patches for registration and respiratory or cardiac movement compensation [2] would be beneficial in the widespread adoption of EMT technology in the operating room.

Much of the previous work in the development of wireless EMT solutions involves sampling the sensor voltage and transmitting the digitised signal over a wireless radio link. The wireless tracking system developed in [3] used three variously oriented magnetic sensors to determine the device position and orientation, with a device diameter of 3.36 cm. The requirement for a 3-axis magnetic sensor does not lend itself to further miniaturisation of the sensor module. A wireless and battery-less device tracked using magnetic gradient fields achieved 0.1 mm accuracy [4]. However, the device has a width of 6.5 mm making it unusable in applications such as bronchoscope tracking [5], where the working channel is typically less than 3 mm in diameter. Another such device was developed based on frequency division multiplexing magnetic localization achieving an accuracy of less than 1 mm [6]. The tracking frequencies used are in the MHz-range, which will likely cause significant distortion from the secondary fields generated by high-frequency eddy currents induced in conductive materials near the tracking volume.

Alternatively, wireless tracking of passive inductor–capacitor (LC) resonant magnetic markers has been demonstrated in both academic settings [7] and in commercial systems such as the Calypso system from Varan Medical Systems Inc (Palo Alto, CA, USA) [8]. Tracking of wireless passive transponders is useful for direct organ target tracking but not currently suitable for instrument-tip tracking applications. Additionally, this tracking method does not allow for tracking of more than approximately five sensors simultaneously.

Frequency modulation (FM) has been previously used to track a wireless capsule endoscope by passing an FM signal directly through the body tissue [9]. However, in the present work it is envisioned that the FM transmitter will be located outside the body, such as integrated in an endoscope handle or on skin-mounted tracking devices.

In FM, the baseband signal modulates the frequency of a high-frequency carrier wave for wireless radio transmission. Encoding the sensor-signal on a frequency modulated carrier signal enables wireless radio frequency transmission of the signal back to the control unit for position and orientation solving. This work introduces a method to wirelessly track an electromagnetic (EM) sensor using an existing EMT system and field generator (Anser EMT, *University College Cork, Ireland*) [10] with no adaption to the system hardware required. The signal from a five-degree-of-freedom (5-DOF) inductive sensor is wirelessly transmitted using a prototype FM transmitter and receiver. The resolved position and orientation accuracy and precision is compared to a typical wired sensor setup and to a gold standard optical tracking system.

## Methods

FM sensor-signal transmission was tested using a multi-channel wireless audio transmitter and receiver (WA-TBT-03S, WA-TBR-03S, *Circuit Design Inc, Nagano, Japan*) with both receiver and transmitter PCBs measuring 70 mm by 80 mm. The RF transmit power was set to 5 mW and the transmit frequency was set to 863.125 MHz with a frequency deviation of 20 kHz.

The Anser EMT system previously developed at University College Cork [10] was used in this work. The Anser EMT system uses frequency division multiplexing to create a spatially unique and time-varying electromagnetic field from eight coils in the field generator (FG) at different frequencies. The sensor pose is solved by minimising the error between the analytical field model (using the Biot-Savart law) and the measured fields. The system is calibrated to account for the physical parameters of the field generator, coil frequencies, sensor, and acquisition channel. The field generator coil frequencies were set in the range 2 to 3 kHz to ensure the FM transmitter and receiver pair operated within the supported audio frequency band.

When placed in the tracking volume of the field generator, the inductive sensor coil produces a time-varying differential voltage signal on the order of micro-volts and must be conditioned before modulation and transmission by the FM radio. The signal was first amplified with a low-noise instrumentation amplifier (INA819, *Texas Instruments, Dallas, TX, USA*) with gain set to 500. The signal was then filtered by an active two-stage 4th-order Butterworth filter with a bandwidth of 10 kHz to remove high-frequency noise before frequency modulation and wireless transmission [11]. The amplifier and filter were implemented on a custom PCB (measuring 50 mm by 90 mm) designed to act as a general analogue front-end (AFE) for inductive sensors in EMT applications. The output from the AFE was connected to the FM transmitter PCB where the signal is frequency modulated and wirelessly transmitted to the FM receiver.

**Fig. 1:**
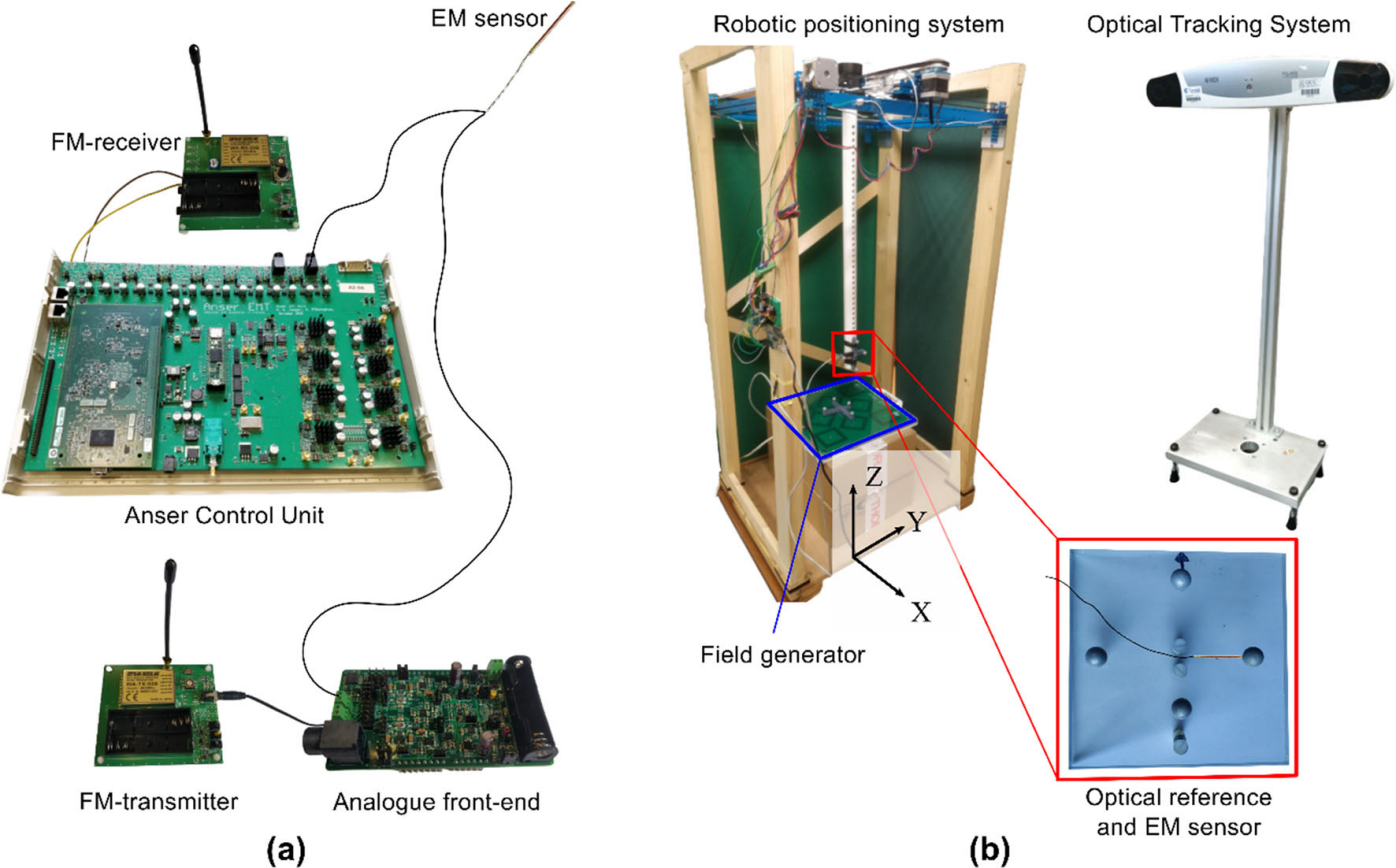
**a** The EM sensor was connected in parallel to the Anser control unit for wired sensor sampling and to the AFE for signal conditioning before FM transmission. The FM receiver was connected to a separate Anser sensor channel for simultaneous wired and wireless sensor sampling. **b** The robotic positioning system was used to collect five grids of test points with the EM sensor shown in the milled slot on the optical reference tool

An EM sensor measuring 0.45 mm in diameter and 8.2 mm in length (610,158, *Northern Digital Inc., Waterloo, Canada*) was connected to the AFE. The sensor-signal was also connected directly to the Anser control unit using two twisted-pair wires (0.1 mm diameter) for comparison. The FM receiver performs demodulation, and the signal was then connected to an input channel on the Anser control unit. Both the directly wired and FM transmitted signals were sampled by the Anser control unit at 100 kHz. This test setup, shown in Fig. 1, allows for simultaneous sampling of the wireless and wired signals from the same sensor.

An optical tracking system (Polaris Vega*, Northern Digital Inc., Waterloo, Canada*) was used as a gold standard for the sensor position. The EM sensor was mechanically fixed in a slot milled on a custom-built optical tool with four passive markers and oriented in the X-axis of the field generator, defined in Fig. 1. A second custom optical tool was attached to the field generator to align the EM and optical tracking reference frames. The linear transformation between the optical tracking and EM reference frames was obtained using a Polaris passive probe stylus (8,700,340, *Northern Digital Inc., Waterloo, Canada*) to perform a pivot calibration.

The sensor was moved using a custom-built robotic positioning system in five grids composed of 5-by-5 test points at heights of approximately 10, 15, 20, 25, and 30 cm above the field generator to give a total of 125 test points. The robot gantry was made from wood and plastic to keep the measurement volume free from metal.

The precision and accuracy of the wired and FM transmitted sensor positions were measured by recording 100 samples at each of the 125 test points. The precision (describing the jitter observed at a stationary position) for each location was determined as the root mean square (RMS) of the Euclidean distance between each of the 100 sample positions and the average position of those samples. The accuracy at every test point was determined as the Euclidean distance between the average resolved sensor position and the optical reference. The global accuracy is reported as the average error over all test points and the standard deviation of those errors. Orientation error is defined as the angle between the optical reference and the resolved sensor axis direction. The mean and standard deviation of the orientation error compared to the optical tracking system are also reported for the wired and wireless cases.

## Results

**Fig. 2:**
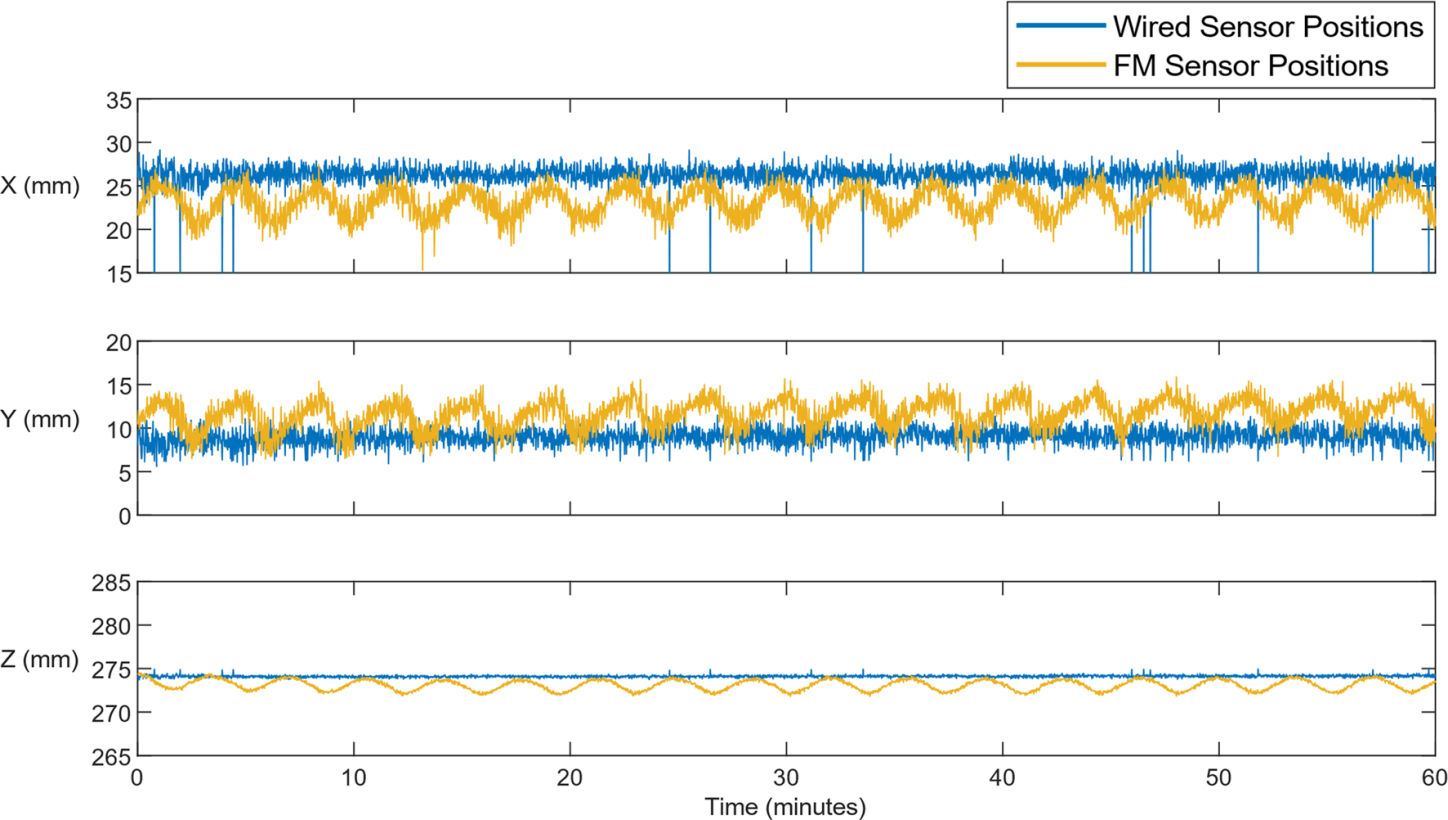
X, Y, and Z resolved positions for a stationary sensor. The sensor was wired in parallel to the Anser base station and wirelessly transmitted using the FM transmitter and receiver prototype

A very low-frequency oscillation in the field strengths with the sensor at a stationary position was observed in the case of the FM transmitted signal. This oscillation had a period of 215 s (frequency = 5 mHz) with a maximum variation in the resolved positions of 5 mm, 6 mm, and 2 mm in the X, Y, and Z directions, respectively. This oscillation is not seen in the case of the directly wired signal and is likely an effect introduced by the FM transceiver module used, for which the intended use case is audio transmission. The resolved sensor positions of the wired and FM transmitted signals are recorded for one hour and plotted in Fig. 2.

To test the feasibility of using an FM transmission scheme for wireless EMT and remove the time-dependence of the measurement, the FM signal was scaled by the ratio of the average FM transmitted field strengths to the average wired field strengths for each test point. This compensated for the effect of the oscillation in the prototype hardware while still evaluating the performance of the wireless communication method. The accuracy of the resolved sensor pose using wireless FM transmission (both compensated and uncompensated) is reported in Table 1, along with wired and wireless orientation errors.

**Table 1:** Mean and standard deviation of position accuracy and orientation error over the 25 points in each grid height for the wired and wireless tests. Positional accuracy of both the low-frequency oscillation-compensated and uncompensated cases are reported for the wireless case only. The accuracy of the wired sensor was the same as the compensated FM transmitted position for all grid heights and is excluded for brevity

Grid height (mm)	Accuracy, uncompensated (mm)	Accuracy, compensated (mm)	Wired orientation error (°)	Wireless orientation error (°)
100	2.92 ± 1.17	2.68 ± 1.11	0.01 ± 0.01	0.06 ± 0.06
150	1.82 ± 0.75	1.62 ± 0.60	0.01 ± 0.01	0.04 ± 0.03
200	1.65 ± 0.71	1.18 ± 0.46	0.01 ± 0.01	0.04 ± 0.02
250	2.43 ± 1.21	1.25 ± 0.39	0.02 ± 0.01	0.03 ± 0.02
300	2.35 ± 0.82	1.32 ± 0.60	0.02 ± 0.01	0.03 ± 0.02
Global average	2.24 ± 0.96	1.61 ± 0.68	0.01 ± 0.01	0.04 ± 0.04

**Fig. 3:**
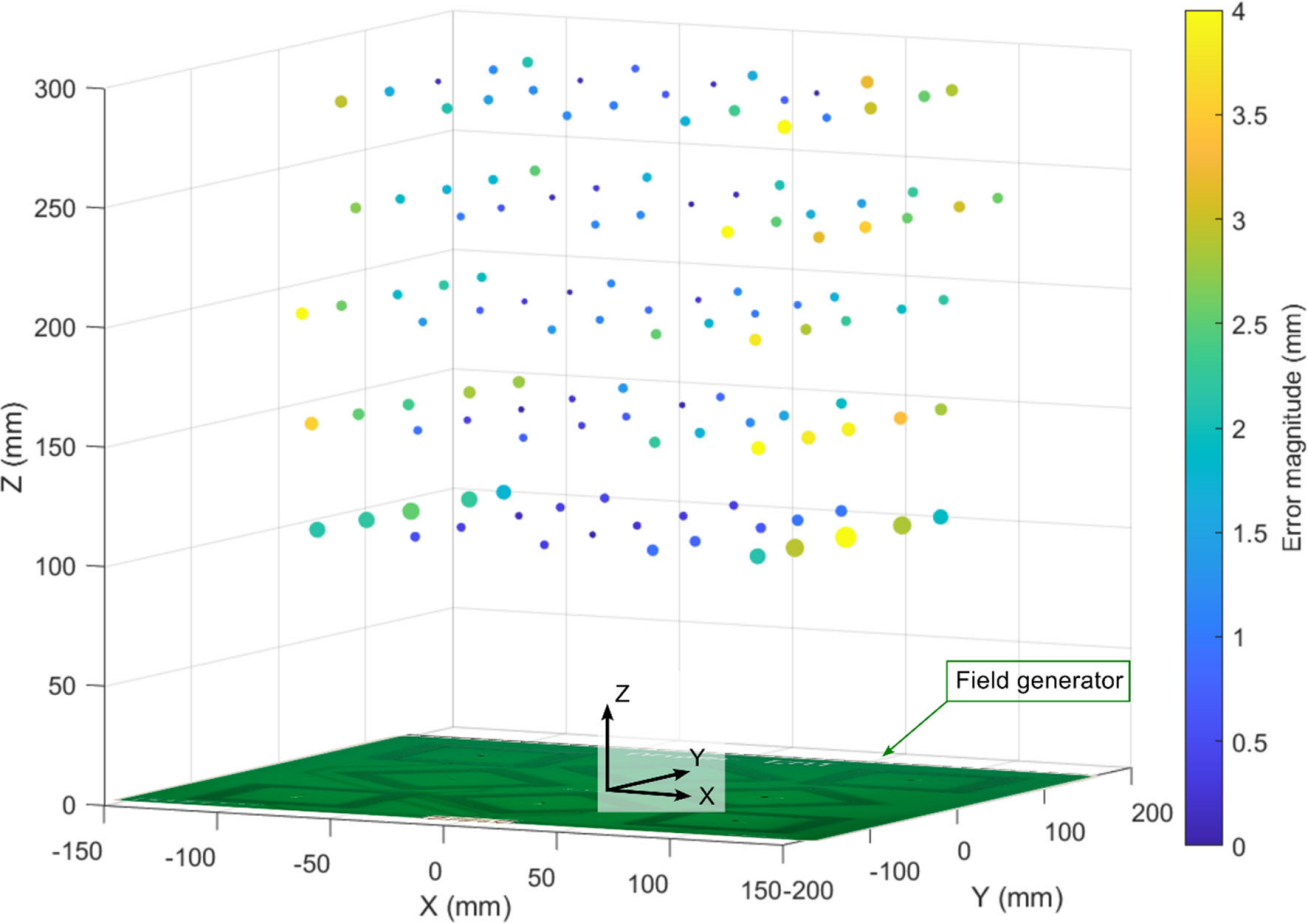
Magnitude of average error in resolved wireless sensor position at each of the 125 test points with their locations shown with respect to the field generator

The average accuracy over all five grid heights was 2.24 ± 0.96 mm and 1.61 ± 0.68 mm for the uncompensated and compensated wireless cases, respectively. The worst pose error over all 125 test points was 6.76 mm and 0.19°, located in the 100 mm grid at the edge of the tracking volume. The error magnitude of each resolved position from the FM transmitted signal with respect to the optical tracker reference position is shown in Fig. 3.

**Fig. 4:**
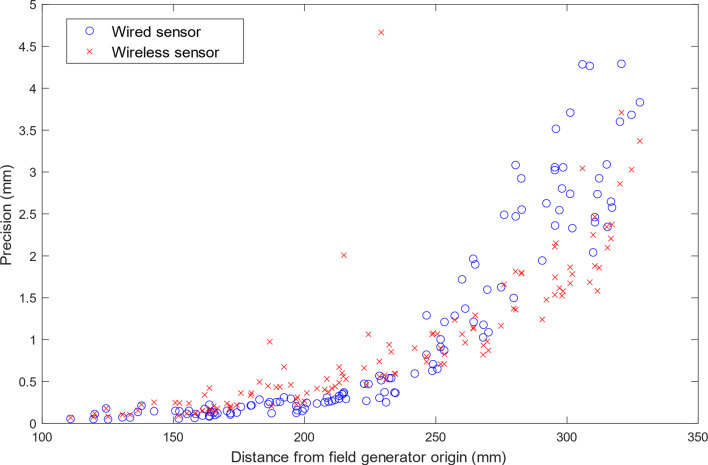
Precision of resolved sensor position calculated from 100 position measurements at each of the 125 test points

The average precision over all test points was 1.09 ± 1.21 mm and 0.95 ± 0.86 mm for the wired and wireless transmitted signals, respectively. The precision in both cases was found to decrease with distance from the origin of the field generator, as seen in Fig. 4.

## Conclusions

The accuracy of the FM transmitted signal without correcting for the field oscillations was 2.24 mm, suggesting that FM transmission is a viable solution to accomplishing wireless EMT. Orientation errors were also acceptable for clinical applications, with all orientation errors below 0.2°. The signal modulation, wireless transmission, and subsequent demodulation did not significantly distort the resolved pose of the sensor. When the effect of the field oscillations was accounted for, the accuracy of the resolved position of the FM signal was 1.61 mm. After this compensation was applied, the resolved position of the FM transmitted signal was the same as the directly wired signal.

An accuracy of 1.14 ± 0.80 mm was previously reported for the Anser EMT system [10]. The mean accuracy of the Aurora planar field generator (*Northern Digital Inc., Waterloo, Canada*) was found to be 0.80 mm in a grid 5 cm above the field generator and 4.30 mm at a height of 30 cm [12]. The average wireless system accuracy of 1.61 mm reported in this work shows that using wireless FM transmission has minimal effect on accuracy in comparison with other systems.

The distribution of errors in resolved position are observed to be systematic and greatest around the extremities of the tracking volume, consistent with results previously reported in[13], as seen in Fig. 3. Such systematic errors may be reduced by creating a full magnetic field map of the tracking volume using a robot for automated sampling, rather than relying on the Biot-Savart law to provide an ideal theoretical model of the magnetic field.

The precision was higher for the FM transmitted signal than in the directly wired case. The wired signal is filtered with a passive low-pass first-order filter [10] before sampling on the Anser control unit, while the wireless signal is filtered with an active low-pass 4th-order filter on the AFE PCB. This additional filtering reduces the noise on the wireless signal, resulting in a higher signal-to-noise ratio (SNR) and better precision. The dependence of precision on distance from the field generator origin seen in Fig. 4 is explained by the reduced magnetic field strength and induced sensor voltage leading to a lower SNR as the sensor moves further from the field generator.

This work demonstrates the feasibility of accomplishing wireless EMT using an FM transmission method as opposed to previous works involving digital-based communication methods or passive resonant frequency transponder tracking. The main advantage of using the method presented here is the backwards compatibility with pre-existing EMT systems. FM transmission for wireless EM tracking has the disadvantage of higher power consumption and larger circuit area requirements than other wireless communication technologies such as Bluetooth. This may make the development of miniature battery-powered FM-wireless EM sensors difficult. Nevertheless, significant size reduction may still be achieved by using an integrated circuit FM transmitter rather than the prototype module used. The AFE in this work included two identical sampling channels and other redundant circuitry used in prototyping. The actual area of the critical components in the analogue signal chain is less than 25 mm by 10 mm on a single side of the PCB. Using more advanced manufacturing methods such as two-side assembly and flexible PCBs will enable the design of a wireless transmitter located in an endoscope or bronchoscope handle with an EM sensor embedded in the distal tip of the device.

Future work will involve the development of an integrated FM transmitter sensor module compatible with the Anser EMT system or the NDI Aurora system (*Northern Digital Inc., Waterloo, Canada*) enabling real-time wireless tracking of surgical instruments.
